# A trusted node–free eight-user metropolitan quantum communication network

**DOI:** 10.1126/sciadv.aba0959

**Published:** 2020-09-02

**Authors:** Siddarth Koduru Joshi, Djeylan Aktas, Sören Wengerowsky, Martin Lončarić, Sebastian Philipp Neumann, Bo Liu, Thomas Scheidl, Guillermo Currás Lorenzo, Željko Samec, Laurent Kling, Alex Qiu, Mohsen Razavi, Mario Stipčević, John G. Rarity, Rupert Ursin

**Affiliations:** 1 Quantum Engineering Technology Labs, H. H. Wills Physics Laboratory and Department of Electrical and Electronic Engineering, University of Bristol, Merchant Venturers Building, Woodland Road, Bristol BS8 1UB, UK.; 2 Institute for Quantum Optics and Quantum Information–Vienna (IQOQI) and Vienna Center for Quantum Science and Technology (VCQ), Vienna, Austria.; 3 Photonics and Quantum Optics Research Unit, Center of Excellence for Advanced Materials and Sensing Devices, Ruđer Bošković Institute, Zagreb, Croatia.; 4 College of Advanced Interdisciplinary Studies, NUDT, Changsha 410073, China.; 5 School of Electronic and Electrical Engineering, University of Leeds, Leeds LS2 9JT, UK.; 6 Quantum Engineering Centre for Doctoral Training, Centre for Nanoscience and Quantum Information, University of Bristol, Bristol, UK.

## Abstract

Quantum communication is rapidly gaining popularity due to its high security and technological maturity. However, most implementations are limited to just two communicating parties (users). Quantum communication networks aim to connect a multitude of users. Here, we present a fully connected quantum communication network on a city-wide scale without active switching or trusted nodes. We demonstrate simultaneous and secure connections between all 28 pairings of eight users. Our novel network topology is easily scalable to many users, allows traffic management features, and minimizes the infrastructure as well as the user hardware needed.

## Teaser

An eight-user fully connected and scalable city-wide quantum communication network can be a basis for the future quantum internet.

## INTRODUCTION

Quantum communication networks present a revolutionary step in the field of quantum communication (*1*, *2*). Despite real-world demonstrations of quantum key distribution (QKD) (*3*–*8*), the difficulty of scaling the standard two-user QKD protocols to many users has prevented the large-scale adoption of quantum communication. Thus far, quantum networks relied on one or more problematic features: trusted nodes (*9*–*13*) that are a potential security risk; active switching (*14*–*17*), which restricts both functionality and connectivity; and, most recently, wavelength multiplexing (*18*) with limited scalability. The ultimate goal of quantum communication research is to enable widespread connectivity, much like the current internet, with security based on the laws of physics rather than computational complexity. To achieve this, a quantum network must be scalable, must allow users with dissimilar hardware, must be compatible with traffic management techniques, must not limit permitted network topologies, and, as far as possible, must avoid potential security risks like trusted nodes.

So far, all demonstrated QKD networks fall in three broad categories. The first category is trusted node networks (*9*–*12*) where some or all nodes in a network are assumed to be safe from eavesdropping. In most practical networks, it is rare to be able to trust every connected node. Furthermore, such networks tend to use multiple copies of both the sender and receiver hardware at each node, thereby increasing the cost prohibitively. The second category is actively switched or “access networks” where only certain pairs of users are allowed to exchange a key at a time (*19*). Similarly, point-to-multipoint networks are useful in niche applications and have been shown using passive beam splitters (BSs) (*20*–*22*), active switches (*14*–*17*), and frequency multiplexing (*17*, *23*–*25*). The last category is fully connected quantum networks, which can be based on high-dimensional/multipartite entanglement to share entanglement resources between several users (*26*, *27*). However, the extreme complexity of changing the dimensionality of the state produced by the source makes this approach unscalable. Fortunately, fully connected networks (i.e., where every user is connected to every other user directly) can be achieved using multiplexing and bipartite entanglement (*18*). Nevertheless, the scheme in (*18*) requires O(*n*^2^) wavelength channels for *n* users, which prevents the technology from being scaled to more than a few users.

Here, we present a city-wide quantum communication network, with eight users, that forms a fully connected graph/network (where each user simultaneously exchanges a secure key with every other user) while requiring only eight wavelength channel pairs, minimal user hardware [i.e., two detectors and a polarization analysis module (PAM)], and no trusted nodes. The quadratic improvement in resources (the number of channels) used is due to passive multiplexing using both wavelength filters and BSs.

Further, to the best of our knowledge, we have demonstrated the largest quantum network without trusted nodes to date. Similar to (*18*), just one source of polarization-entangled photon pairs is shared passively between all users and requires neither trust in the service provider nor adaptations to add or remove users. We performed a full QKD experiment on a city-wide scale in deployed fibers with a mixture of superconducting nanowire single-photon detectors (SNSPDs) and a single-photon avalanche diode (SPAD). Further, we demonstrate a new topology with O(*n*) scaling of all resources consumed using only 16 wavelength channels and 2 BS channels to distribute eight entangled states, among the 28 links, between eight users in a fully connected graph using only one fiber and PAM per user.

## RESULTS

**Fig. 1:**
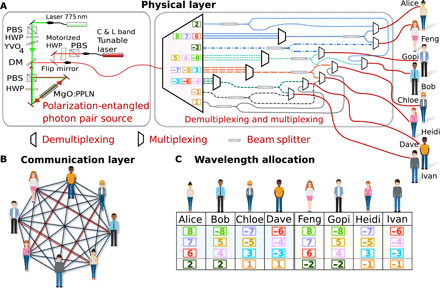
The overall network architecture showing the physical layer, communication layer, and the way wavelength channels are distributed. The network consists of two layers. (**A**) The physical layer contains the source of entanglement (blue) and the multiplexing unit (MU; gray). These form the QNSP. Our topology uses just one deployed fiber (red) per user to interconnect all eight individual users. (**B**) The communication layer forms a fully connected graph without trusted nodes for entanglement distribution, key exchange, and secure communication (classical communication channels between users are not shown). Each line represents a link—the sharing of a bipartite entangled state. Higher-bandwidth links share a second entangled state shown as a red line. (**C**) Wavelength allocation: Every user of the eight-node network receives four wavelength channels denoted by a number (which corresponds to their ITU 100-GHz DWDM grid channel number minus 34). That is, ITU channel 34 (or 0 in the figure’s naming convention) corresponds to the channel approximately centered at the down-conversion degeneracy wavelength. Thus, a pair of matching colors or numbers with equal absolute values and opposite sign denote wavelengths corresponding to an entangled photon pair. The regions shaded in blue and yellow are identical subnets and represent the multiplexing using BSs. The last row below the dotted line shows the additional wavelength channel needed to fully interconnect the two subnets. Certain user pairs are connected by two entangled photon pairs (such as Alice and Gopi via {8, −8} and {2, −2}) and consequently enjoy an increased key rate.

**Fig. 2:**
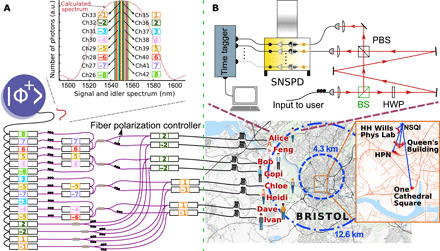
The experimental setup showing the multiplexing and demultiplexing steps along with the user module and distribution of users across the city of Bristol. A source of bipartite polarization-entangled photon pairs with a broadband signal and idler spectrum [as shown in the inset in (A)] produces a ∣Φ^+^〉 Bell state that is wavelength and BS multiplexed, as shown. Wavelength multiplexing was performed using 100-GHz International Telecommunication Union’s (ITU) DWDM channels, represented as colored numbers plus or minus the central channel 34. BS multiplexing used 50:50 fiber BS. Photons were sent to each user via loop-backs from deployed fibers spread across the Bristol city center (blue links in the above map) or several kilometers of fiber coil whose effective coverage is shown by the blue dashed circles on the map. The measurement apparatus of each user is shown in the inset in (B). To the left of the dashed green line is the QNSP. Maps were plotted using data from mappyplace.com and mapiful.com. (**A**) The spectrum of the signal and idler photons was calculated from datasheet values and Sellmeier coefficients of (*34*). Energy conservation ensures that pairs of wavelengths, when at the same spectral distance from the central wavelength, are correlated. Such a pair of wavelength channels is indicated by the same color number with and without the minus sign. The ITU channel numbers along with their representative colored numbers are shown. (**B**) The PAM of each user consists of a BS to direct input photons along either the short or long optical path. The short path measures the polarization in the HV basis using a polarizing beam splitter (PBS) and two SNSPDs. The long path includes an achromatic half–wave plate (HWP) to rotate the measurement basis to DA and measures using the same PBS and SNSPDs. a.u., arbitrary units.

By using a combination of standard telecom dense wavelength division multiplexers (DWDM) with 100-GHz channel spacing and BS multiplexing using in-fiber BSs, we were able to distribute bipartite entangled states between all users from just one source of polarization-entangled photon pairs. The network architecture requires only 16 wavelength channels to fully interconnect eight users, as opposed to the 56 channels that would be necessary following our earlier scheme (*18*). The network architecture is best understood when divided into different layers of abstraction, as shown in Fig. 1. The bottom “physical layer” represents the actual infrastructure that supports the network and comprises a central quantum network service provider (QNSP) and the user hardware connected to the QNSP via distribution fibers. In the physical layer, the network topology requires only one fiber between each user and the service provider, while in the logical/connection layer, the topology naturally forms a fully connected graph between all 28 unique pairs formed by eight users (see Fig. 1). Every user is equipped with a PAM that implements a passive basis choice using two single-photon detectors each, as shown in Fig. 2. Users can demultiplex the incident photons such that each detector receives fewer wavelength channels to improve their signal-to-noise ratio and therefore the key rate. We generate secure keys between all 28 links formed by pairs of users. Four of these links can be chosen to have premium connections with increased key rates, which, when combined with active switching, can provide traffic management on the network. Last, we demonstrate that our network is capable of supporting a mixture of both SNSPD- and SPAD-based user platforms.

The details of the QNSP are shown to the left of Fig. 2. It consists of both the source of polarization-entangled photon pairs and the multiplexing unit (MU) comprising WDMs and BSs. All multiplexing is performed in a single MU, colocated with the source in our implementation. To take advantage of existing fiber infrastructure, channels for many users can be sent along fewer fibers, and multiple MUs, at various locations closer to clusters of users, can be used to create this quantum network. The user hardware—a PAM and two single-photon detectors—is shown in Fig. 2B (inset). Photons incident on a PAM are directed by a BS along either the short path where they are measured in the horizontal/vertical (HV) polarization basis or the long path and through a half–wave plate (HWP) such that they are measured in the diagonal/antidiagonal (DA) polarization basis. The overall result is that the physical layer constitutes a relatively simple hub and spoke topology, while in the logical layer, every pair of users always share an entangled photon pair.

We conceptually divide the eight users of our network referred to as Alice (A), Bob (B), Chloe (C), Dave (D), Feng (F), Gopi (G), Heidi (H), and Ivan (I) into two subnets of four users (see Fig. 1). A subnet uses wavelength multiplexing to form a fully connected network among its members—A, B, C, and D. BSs are then used in each of the wavelength channels to duplicate the first subgroup creating a second set of four interconnected users—F, G, H, and I. Thus, entanglement is shared between every pair of users except AF, BG, CH, and DI as the above splitting also gives rise to connections between the two sets. Two additional wavelength pairs are then distributed between these pairs of users to create the fully connected network of eight users with only 16 wavelength channels. Each pair of users performs a standard BBM92 (*28*) protocol where the photons shared with all other users are treated as background noise. A narrow coincidence window, optimized in postprocessing and typically about 130 ps, ensures that this noise only contributes minimally to the quantum bit error rate (QBER).

All multiplexing and demultiplexing in the experiment is performed with standard telecommunications equipment. The experimental setup (shown in Fig. 2) uses a broadband source of polarization-entangled photon pairs at telecommunications wavelengths similar to that described in (*18*). Comparable sources have also been reported in (*19*, *23*, *25*, *29*, *30*) where a ≈775-nm pump down-converts in a type 0 MgO:PPLN (Periodically Poled Lithium Niobate) crystal to produce signal and idler photons with a full width at half maximum bandwidth of ≈60 nm centered at 1550.217 nm [roughly corresponding to the International Telecommunication Union’s (ITU) 100 GHz DWDM grid at channel 34 (Ch34) centered at 1550.12 nm]. Because of energy conservation during the down-conversion process, only frequencies equidistant from half the pump frequency can support entangled pairs. Thus, wavelengths corresponding to the channel pairs {Ch33, Ch35}, {Ch32, Ch36}, {Ch31, Ch37}, and so on are entangled with each other. The wide signal and idler spectra were demultiplexed into eight wavelength pairs, as above, and each user is given a combination of wavelengths according to the table in Fig. 1. Thus, each user receives four wavelength-multiplexed channels simultaneously via one single-mode fiber, and eight different entangled states are shared between the 28 different pairs of users.

**Fig. 3:**
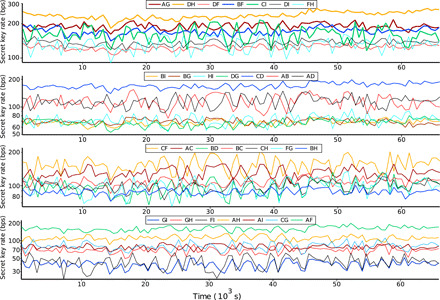
Secure key rate over time for the laboratory experiment. A secure key was generated every 10 min while including finite key effects and a security parameter of 10^−5^. The length of each link is given in table S3, while the average secure key rate for each link is tabulated in table S2 for the metropolitan network and in table S1 for the in-laboratory demonstration.

The experiment was performed in two stages. In the first stage, the QNSP, the MU, the eight users each connected to the QNSP/MU with a single fiber ≈10 m in length, and the 16 detectors were situated in a single laboratory in the Nano Science and Quantum Information building in Bristol. To demonstrate the stability of our network, we recorded data for 18.45 hours, as shown in Fig. 3. To be able to account for finite key effects with a security parameter of 10^−5^, we computed the private key once every 10 min, and the figure shows the average secure key generation rate per second in each 10-min period for each of the 28 links (discussed further in Materials and Methods). The total secure key obtained is shown in table S1. Users A through H used superconducting nanowire detectors from Photon Spot, while Ivan used a combination of one SNSPD and one InGaAs SPAD. We note that the use of heterogeneous detectors did not substantially affect the key generation rates.

In the second stage, the connection between the user and the MU was replaced for six users by long-distance links. Furthermore, the SPAD was exchanged between Ivan and Gopi. Alice was connected via a 12.6-km spool with a loss of 13.3 dB, Chloe was connected to a loop-back from a laboratory in the first floor of the physics building of the University of Bristol with a total distance of 463 m and 1.36-dB loss, Dave used a 4.3-km spool (15.7 dB), Feng looped back from the basement of the Merchant Venturers Building through 1.625 km of deployed fiber (and a loss of 2.04 dB), Heidi used a loop-back connection from the ground floor of Queen’s Building with a loss of 1.68 dB and a total distance of 1.624 km, and Ivan was connected to another loop-back from the server room of One Cathedral Square in the city center totaling 3.10 km (2.57 dB). Bob and Gopi continued to be connected via short fibers. Thus, the 28 links varied in the effective separation of users from 16.6 km to ≈10 m. This shows the versatility of the network architecture as both a local area network and a city-wide metropolitan area network. Table S2 shows the secure key rate over these long distances in deployed fiber and in fiber spools.

The QBER, and hence the secure key rate, in our proof-of-principle experiment was limited by two main experimental imperfections: First, a more careful fiber neutralization procedure using the manual fiber polarization controllers (FPCs) would significantly improve the QBER. Second, the alignment of the HWP and the extinction ratio of the polarizing BS (PBS) used in each user’s PAM could be further optimized (see the Supplementary Materials).

Further optimization of the secure key rate is possible by adjusting the pump power in the source, thus changing the pair generation rate (see fig. S2). We cannot adjust the pair generation rate in each pair of wavelength channels separately. Thus, the optimum pump power is strongly affected by the different types/alignment of user hardware (like detectors and PAMs) in the network.

Nevertheless, the measured QBER proved to be stable in an 18.45-hour laboratory test (see fig. S3) and resulted in a stable and positive overall secure key rate in a 7-hour metropolitan quantum communication network demonstration (see fig. S4).

**Fig. 4:**
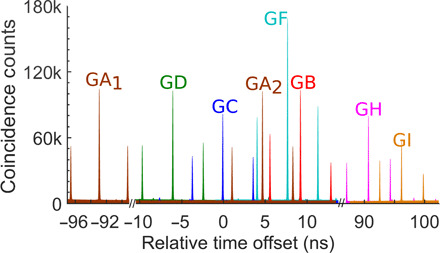
Temporal cross-correlation histograms between Gopi and all other users. Each pair of users identify photon pairs by their arrival time using *g*^(2)^ histograms. The data shown here were collected over 1 hour for user Gopi (G) during the laboratory demonstration of the network. Users G and A share two sets of correlated wavelengths to enable higher key rates; therefore, they share two sets of *g*^(2)^ peaks (GA_1_ and GA_2_). Information as to which detector(s) clicked was obscured by all users. Figure S4 shows the histograms between each pair of detectors for the users G and B; however, this more detailed graph contains information about the measurement outcome and cannot be used to generate a key.

Using a low-cost design for the PAM at each node, any pair of communicating users (say Alice and Bob) obtain three peaks in their temporal cross-correlation histogram *g*^(2)^ between each detector of A and B for each correlated pair of wavelengths they share (see Fig. 4 and fig. S1). Under normal operation in the absence of an eavesdropper, the central peak corresponds to all measurements where A and B chose the same measurement basis, while the side peaks correspond to A and B choosing different measurement bases. Since we do not explicitly note down the basis choice, we must assume that the BS ensures that we measure in both necessary measurement bases. In general, it is sufficient to assume that the BS has a bound on its splitting ratio. See the Supplementary Materials for more details on the security.

We note that each user only shares the time of a detection event and not which detector clicked as required by the protocol. Since the two detectors used by a user can have different delays or jitters, every user must characterize their setup and modify the time tags before they are shared.

## DISCUSSION

We have successfully realized a complete entanglement-based quantum communication network with improved scaling, traffic management, and long-distance links via deployed fiber throughout the city. We have shown the effectiveness of a new and improved network architecture. Our fully connected scheme can be modified at the software or hardware level to create any desired subgraph. Further, by multiplexing states intended for several users into a single fiber and demultiplexing them later on, our architecture can easily support any desired complex network (see the Supplementary Materials).

As the number of users increases, the QNSP can choose to use additional wavelength channels, which (up to a limit based on noise counts as discussed in the Supplementary Materials) minimally affects the key rates of all existing users. This detrimental impact can be completely negated by users selectively detecting only the desired wavelengths. Alternatively, to increase the number of users, the QNSP can use additional BSs, which would reduce key rates but drastically increase the number of users on the network with the fewest additional wavelength channels. However, this would irrecoverably affect the key rates of all users. In our network, the physical topology grows linearly with each additional user requiring only one additional PAM and fiber. Photons intended for multiple users can also be multiplexed through the same fiber to optimize cost/convenience of distribution. We note that the network is also capable of producing all possible subgraphs, adding or removing users and changing the allocation of premium connections without altering the source of entanglement.

Detectors are a significant resource for individual users; therefore, we significantly lower the financial cost per user by sending several channels onto the same detector at the expense of a slight increase in QBER. However, this can be mitigated by demultiplexing the signal on each detector to multiple detectors. The all-passive trusted node–free implementation could allow us to use active switching to incorporate additional functionality such as traffic and bandwidth management, software-defined networking, etc. The ≈17-km, or more, range of the network, as we have demonstrated, is more than enough to create local and metropolitan area networks interconnecting end users throughout a city or building. This range can be further extended by repeaters, reduced detector jitter, wavelength demultiplexing to several detectors, and wavelength-selective switching to the same detector.

The number of users that can be connected to a given network is limited by available resources, loss, and the marginal increase in error rate with each simultaneous connection established (with a given detector). The error rate is theoretically limited by the probability that uncorrelated photon detection events can accidentally occur in any given coincidence time window. In terms of resource, the network scales linearly with respect to user hardware and number of deployed fibers. On the service provider’s side, the number of wavelength channels can be increased drastically by using closer-spaced, narrower-band WDMs and by generating broader-band down-conversion. For example, periodically poled fibers are a very promising method of the latter (*30*). Using polarization preserving methods of multiplexing, such as an on-chip design, would eliminate the need for most FPCs. We were able to demonstrate this by connecting several extra kilometers of fiber to many users and maximizing the key rate using only the FPCs on each user’s module instead of the FPCs of the service provider. Further, simulations show that the network topology can be extended to 32 (49) users divided into 2 (7) subnets while maintaining a reasonable secure key rate (see fig. S5).

The long-term goal of a full-fledged quantum internet requires quantum communication networks that support other forms of quantum information processing or other quantum technologies. The vast majority of such applications rely on the distribution of entanglement between several nodes, making the current architecture an ideal candidate for further study.

## MATERIALS AND METHODS

### Quantum network service provider

The network consists of the QNSP, distribution fibers, and users. The QNSP is composed of a photon pair source set up to prepare bipartite polarization-entangled states and a MU.

The source is pumped by diagonally polarized light ∣*D*〉, from a continuous wave (CW) pump laser emitting at 775.1085 nm, which passes through a dichroic mirror and a PBS that defines the input and output of a Sagnac loop (see Fig. 2) (*25*, *31*, *32*). The horizontally (vertically) polarized pump light thus propagates counterclockwise (clockwise) inside the loop. A HWP after the transmission port of the PBS rotates the polarization by 90^∘^ to vertical. This allows for injecting light in both directions of a 5-cm-long magnesium oxide–doped, periodically poled lithium niobate (MgO:PPLN) bulk crystal with a poling period of 19.2 μm in which vertically polarized pump photons are converted to vertically polarized signal and idler photon pairs through type 0 spontaneous parametric down-conversion, i.e., ∣*V*〉 → ∣*V_s_*〉∣*V_i_*〉. The photon pair contribution in the clockwise direction is rotated by the HWP and therefore becomes ∣*H_s_*〉∣*H_i_*〉. Both contributions are then coherently combined at the PBS and are isolated from the pump light by the dichroic mirror. Ideally, this will create maximally polarization-entangled states for each set of two different wavelengths, λ_1_ and λ_2_, located symmetrically about the central wavelength at 1550.217 nm

$$|\Phi^{+}\rangle =\frac{1}{\sqrt{2}}(|V_{\lambda_{1}}V_{\lambda_{2}}\rangle +|H_{\lambda_{1}}H_{\lambda_{2}}\rangle )$$ (1)

The MU consists of off-the-shelf DWM filters. In addition, the MU also has a set of 50:50 fiber BSs. These components were spliced together to distribute photon pairs from the source to each of the eight distribution fibers, as shown in Figs. 1 and 2.

The spatial mode containing the signal and idler photons from the source was coupled into one single-mode fiber and spectrally split by a thin-film DWDM from Opneti (with a channel spacing and a nominal full width of 100 GHz) into 32 channels, as defined by the ITU in G.694.1. Our QNSP consists of one 32-channel DWDM (of which only 16 are used) exhibiting insertion losses per channel between 0.6 and 2.5 dB [and a polarization-dependent loss (PDL) < 2.5 dB according to the datasheet], 16 add/drop DWDMs with ≈0.5 dB insertion loss (PDL < 0.1 dB), and eight standard fused couplers with insertion loss below 3.4 dB (PDL < 0.1 dB). Optical multiplexers form the foundation of DWDM networks deployed by the telecommunication industry. There are currently two main technologies used in the industry, thin-film filters (TFFs) and arrayed waveguide gratings (AWGs). TFFs function by filtering wavelengths serially and are designed to transmit a specific wavelength while reflecting all others. They are made of a concatenation of interference filters each fabricated with a different set of dielectric coating. AWGs are single-stage filters that deploy planar waveguide technology consisting of free propagating regions interconnected by waveguides. The waveguides have different lengths leading to constructive or destructive interferences in the output and thus multiplexing/demultiplexing. Because of their low cost and robustness against thermal fluctuations, we have chosen TFF to build our QSNP. In addition, the main advantage of AWGs over TFF is that the parallel multiplexing approach is more conductive to high channel-count applications, which is not relevant for quantum signal levels.

We selected 16 wavelength channels symmetrically with respect to the degeneracy wavelength of 1550.217 nm, which corresponds to ITU channel 34 (see Fig. 2A, inset). On the red side of the spectrum, we used ITU’s frequency channels 26 to 33 and channels 35 to 42 on the blue side. Because of the well-defined pump wavelength of the CW laser and energy conservation during down-conversion, we obtained polarization entanglement between pairs of channels (26 and 42, 27 and 41, 28 and 40, and so on).

Each of the 16 wavelength channels is then split by a BS, such that, in total, 32 possible pairs of correlated photons are available. Using further add-drop multiplexers before and after the BSs, four channels were combined into each single-mode fiber to every user. Since the partner photons for each channel can be found in two other fibers, each of the users now holds eight polarization-entangled connections to other users. This means that each user is connected to all the other users, featuring one doubled connection. FPCs were used to ensure that the reference frame of polarization in the source is (nearly) identical to that of the PAM.

It was not necessary to compensate all channels independently. Similar wavelengths were compensated together. At the end, each user received four channels (see Figs. 1 and 2) via a single-distribution fiber and used a PAM to measure in the HV or DA polarization basis.

The distribution fibers were all single mode for 1550 nm but of varying lengths and specifications. Several of the fibers were deployed across the university and the city of Bristol. We conducted two experimental runs, the first with short distribution fibers and the second with varying link lengths, as shown in table S3.

### Users

Each user in the network is equipped with the PAM and two single-photon detectors. The PAM enables passive switching between photon polarization measurements in two orthonormal bases (either HV or DA). A BS at the PAM’s input randomly directs incoming photons either through the short optical path to the PBS and measurement in HV basis or through the long optical path with an achromatic HWP, providing a 45^∘^ polarization rotation and the same PBS for measurement in DA basis. The difference between the long and short free-space paths corresponds to 3.7 ns of time delay, resulting in polarization analysis in different time bins (*29*).

We designed the PAM to be completely passive, compact, portable, simple, and cheap to mass produce and align, but still robust and stable. At each PAM’s input and outputs to two detectors, SMF28e single-mode fibers are connected to collimators/couplers with custom-produced 15.7-mm effective focal length (at 1550 nm) SF11 glass singlet lenses (antireflection coated for 1500 to 1600 nm), with *x*-*y*, tip/tilt, and focus adjustment. Cube BS and PBS are mounted on kinematic platforms for rotation and tip/tilt adjustment. The achromatic HWP is mounted in a manual precision rotation mount. The long optical path was realized using unprotected gold mirrors on tip/tilt kinematic mounts.

Commercially available BS, PBS, HWP, and unprotected gold mirrors were used (Thorlabs BS012, PBS104, AHWP05M-1600, and PFSQ10-03-M03). BS characteristics are *T* = (56 ± 8)% and *R* = (44 ± 8)%, depending on input polarization and orientation angle. The PBSs in use have extinction ratios *T_p_* : *T_s_* > 1000 : 1, *R_s_* : *R_p_* roughly between 20:1 to 100:1, transmission efficiency *T_p_* > 90%, and reflection efficiency *R_s_* > 99.5%, where *T* and *R* represent transmitted and reflected ports, respectively, and the subscripts *s* and *p* represent the *s* and *p* polarized components. Achromatic HWP retardance accuracy is <λ/300. Complete production of lenses and optomechanics as well as assembly was done at the Ruđer Bošković Institute, in the optical and mechanical workshops of the Division of Physical Chemistry.

The PAM outputs are fiber-coupled and launched into two single-photon detectors. We used 15 SNSPDs from Photon Spot with detection efficiencies ranging from ≈70 to 90%, a jitter of between ≈60 and 80 ps (including the measurement device), and dark counts of ≈1 kHz and one InGaAs avalanche SPAD from ID Quantique, model ID230, which has 20% efficiency, ≈100 ps jitter, and dark counts of ≈0.05 kHz. After detection, the optical signal is converted into an electronic pulse and read out at an 18-channel time-tagging module (Swabian instrument model Time Tagger Ultra). Using a laptop, we performed an on-the-fly computation of coincidences, basis reconciliation, and secure key rate estimation for all 28 QKD links.

### Secure key generation

Because of the design of PAMs and the CW pump of the source, we only know whether the measurement basis choice (of a pair of users) was matched or not. Here, the information of which detector clicked directly encodes the measurement outcome without revealing the measurement basis choice. Thus, we cannot know the basis in which a detected photon was measured. Suppose the time delay between Alice’s Horizontal/Anti-diagonal (HA) detector to Bob’s HA detector is longer than the delay between Alice’s Vertical/Diagonal (VD) detector and Bob’s VD detector, then by looking at the *g*^(2)^ histogram, Eve can identify two different delays, which means that Eve can guess what the measurement outcome was and thus what the key bit could be. Thus, all users must merge the time tags of all detectors into a single data set without which-channel or which-detector information.

Then, all users exchange their merged time tags with each other via the authenticated public channel(s). After the time tags are shared among all users, they calculate a temporal cross-correlation histogram (*g*^(2)^) to find the coincidences. Users assign “0” or “1” to the measurement outcomes where they both detect a photon within the coincidence window and happened to measure in the same basis.

After obtaining the sifted keys, all users perform the error correction and privacy amplification procedures to extract the final secure key. Assuming that each pair of users has been able to identify and include in their sifted key only those rounds in which they happened to measure in the same basis (see security considerations in the Supplementary Materials for more details), their final secure key length *n_f_* can be calculated by

$$n_{f}\geq n_{s}[1-H_{2}(e_{p}^{U})-fH_{2}(e_{b})]$$ (2)

where *n_s_* is the sifted key length, *e_b_* is the measured QBER, $e_{p}^{U}$ is the estimated upper bound of phase error rate, *H*_2_(*x*) is the binary Shannon entropy, and *f* is the error correction inefficiency (assumed to be 1).

Since we could not divide our sifted key into two individual bases (*Z* and *X*), here we analyze the phase error rate in the mixed basis case. Given failure probability ξ*_ph_*, the upper bound of phase error rate can be estimated by

$$e_{p}^{U}=\alpha e_{b}+(1+\alpha )\sqrt{\frac{\text{ln}\;4-2\text{ln}\;\xi_{\mathrm{ph}}}{n_{s}}}$$ (3)

where α ≥ 1, and the phase error probability is α times bit error probability. In our experiment, we used passive measurement modules with 50:50 BSs to perform unbiased measurement basis choices, which results in α = 1 (*33*). Similar arguments can be made to show security even in the case of bias in the measurement basis choice. From the experimental data, we can infer this bias under the assumption that the fiber coupling for both detectors of the PAM is equal. Assume that the basis choice bias of Alice and Bob is $p_{Z}^{A}$ and $p_{Z}^{B}$, then the coincidence counts of the left histogram peak between a pair of users (as seen in the histograms of fig. S1) are related to $p_{Z}^{A}(1-p_{Z}^{B})$, the coincidence counts of the middle peak are related to $p_{Z}^{A}p_{Z}^{B}+(1-p_{Z}^{A})(1-p_{Z}^{B})$, and the coincidence count of the right peak is related to $(1-p_{Z}^{A})p_{Z}^{B}$. Therefore, when the basis choice is biased, one could first measure $p_{Z}^{A}$ and $p_{Z}^{B}$ by monitoring the coincidence counts in the left, middle, and right peaks. Then, one can estimate α using these values of $p_{Z}^{A}$ and $p_{Z}^{B}$. More details on the security of this scheme can be found in the Supplementary Materials.

## Supplementary Material

aba0959_SM.pdf

## SUPPLEMENTARY MATERIALS

Supplementary material for this article is available at http://advances.sciencemag.org/cgi/content/full/6/36/eaba0959/DC1
