# Supplementary material for: A trusted node–free eight-user metropolitan quantum communication network
Source: Sci Adv. 2020 Sep 2;6(36):eaba0959. doi: 10.1126/sciadv.aba0959 (PMC7467697; doi:10.1126/sciadv.aba0959)
Supplement: aba0959_SM.pdf [file aba0959_SM.pdf]

Science Advances
AAAS

advances.sciencemag.org/cgi/content/full/6/36/eaba0959/DC1

Supplementary Materials for

A trusted node–free eight-user metropolitan quantum communication network

Siddarth Koduru Joshi*, Djeylan Aktas, Sören Wengerowsky, Martin Lončarić, Sebastian Philipp Neumann, Bo Liu, Thomas Scheidl, Guillermo Currás Lorenzo, Željko Samec, Laurent Kling, Alex Qiu, Mohsen Razavi, Mario Stipčević, John G. Rarity, Rupert Ursin

*Corresponding author. Email: joshi@bristol.ac.uk

Published 2 September 2020, *Sci. Adv.* **6**, eaba0959 (2020)
DOI: 10.1126/sciadv.aba0959

This PDF file includes:

Supplementary Materials and Methods
Tables S1 to S3
Figs. S1 to S5
References

Supplementary Material and Methods

Scalability: We have demonstrated an eight-user quantum communication network enabled by a single source and wavelength/BS multiplexing. The number of wavelength channels available depends on the available WDM technology and the bandwidth of the polarization entangled photon pair source. The current source has a bandwidth of ~60 nm (~7492 GHz) and the WDM channels used have a ~0.8 nm (100 GHz) bandwidth. This limits us to a maximum of ~75 wavelength channels. However, broader bandwidth sources (such as super-continuum based sources of polarization entanglement) and or closer spaced WDM channels (such as the upcoming 10 GHz DWDM standard) would allow at least a few hundred channels.

Thus the real limit to the maximum number of users in such a quantum network is the QBER, specifically the contribution from “accidentals”. Given a particular photon flux incident on the detectors of two users, there is a probability that two uncorrelated detection events happen to occur within the chosen coincidence window. Such coincidences are called accidentals. In our experiment, a user opens a different coincidence window for every incident wavelength channel as needed because of the uncompensated propagation times of different wavelengths via the fibers and DWDM channels. Thus increasing the

number of wavelengths a single user receives increases the accidental rate and the QBER, effectively reducing the secure key rate. The QNSP can correct for the propagation delays between different wavelength and BS, when all users are connected by fixed lengths of fiber, dramatically increasing the secure key rates because each user will then need to consider only a single coincidence window. Similarly the users have two strategies to increase their secure key rates: use multiple detectors for each measurement outcome, each of which detects fewer wavelength channels; or selectively choose which wavelengths are incident on their detectors based on the connection(s) desired with select other users. In the absence of such methods, minimizing the number of multiplexed channels is the best way to increase secure key rates.

By using k-fold beamsplitters, we can create $\frac{n}{k}$ subnets between n users. Each subnet forms a fully connected network with WDMs only using $\frac{n}{k}\left(\frac{n}{k}-1\right)$ wavelength channels. Additional wavelength channels are needed to interconnect the different subgroups. This can be done in two ways: First, we treat each subnet effectively as a single user in a k user network and create a fully connected network with $k(k-1)$ additional wavelength channels. This implies using $\frac{n}{k}$-fold beamsplitters to distribute each of these extra $k(k-1)$ channels to all $\frac{n}{k}$ users in a subnet. When considering the link between just two users, beamsplitters can be viewed as losses. Thus mixing k-fold and $\frac{n}{k}$-fold beamsplitters can result in significantly different coincidence rates between different sets of channels/users. Consequently, the optimal pump power (i.e. pair rate emitted by the source) will be different for various channels. With a single source,

we cannot optimist this independently which in turn leads to sub optimal key rates. Thus in the second method, we can impose the constraint that all wavelength channels are split using only k-fold beamsplitters. Here, each subnet requires $\frac{n}{k^2}$ wavelengths and the network requires a total of $\frac{n}{k}\left(\frac{n}{k}-1\right)+\frac{n}{k^2}k(k-1)$ wavelength channels. We note that the above formulas are valid only when n, k, $\frac{n}{k}$ and $\frac{n}{k^2}$ are all integers. To create networks with any integer number of users, it is possible to create a larger network that satisfies the above conditions and not connect all users as and how required.

With a fixed pump power of the entangled photon source, the net effect of BS multiplexing a correlated wavelength pair is akin to "time sharing" the key. When considering just two users, it can be thought of as additional loss. On the other hand, wavelength multiplexing introduces a new source of key. Both techniques adversely affect the signal to noise ratio however, the additional noise due to more wavelength channels can be avoided by filtering. Thus a good topology makes use of a significant amount of wavelength multiplexing supplemented by BS multiplexing. Fig. **S5** shows the secure key rate for different numbers of users as a function of the transmission loss. For convenience, we have focused on the use of 2-fold beamsplitters (solid lines) and networks where $n = k^2$, using k-fold beamsplitters and k subnets (dashed lines). Maintaining a reasonable secure key rate, within the constraints of the current experiment, is possible for both 32 users in 2 subnets and even 49 users in 7 subnets. This demonstrates how our network architecture can be used for very large and complex Local Area Quantum Networks.

Adapting the network for practical use-cases: In our proof of principle demonstration, the source of polarization entangled photon pairs was adjacent to the Multiplexing Unit (MU) that distributed these pairs among the users. The users were in turn each connected to the MU by single long fibers. In a real world scenario, the MU need not be adjacent to the source nor must the MU be a single unit. Photons from the source can be split and combined in any physical location or in several locations to take advantage of available fiber infrastructure. In our experiment the source was separated from the MU by a 8 m long fiber and each component in the MU was separated by between 2 and 7.5 m of optical fiber.

Further, our network topology allows the flexibility needed for a wide variety of use-cases. Longer distance, or higher speed links can co-exist on the same network; these can be implemented by using multiple detectors on certain nodes where each detector measures some of the incident wavelengths. Access networks are often preferred for low bandwidth use-cases, however users sacrifice anonymity when they request the Quantum Network Service Provider (QNSP) to establish a connection with another chosen user. Here, our topology is capable of supporting an anonymous access network controlled by each user instead of the QNSP.

Optimizing the key rate: It is possible to significantly improve the key rate beyond what is shown in the main paper in several ways. First, increasing the pump power increases the number of photon pairs. Given the detector jitter,

losses and QBER there exists an optimum pump power at which the secure key rate (in bits per second) is maximized. Fig. **S1** shows the key rate of all 28 links in the network measured at 9 different pump powers. Note that when the photon flux is excessive, a secure key cannot always be generated. This is because of the increased contribution of uncorrelated singles to the QBER via accidental counts. Reducing the detector jitter is thus the best way to further increase the key rates. Using a single source for the entire network limits our control over the individual pair production rates for each correlated wavelength pair. Thus using different types of detectors strongly influences the optimal pump power. In addition to different detectors, the alignment of individual PAMs of each user and the FPCs contribute to the overall network performance. Second, using a pulsed pump as discussed in the supplementary material of Ref (18) would help reduce the QBER and significantly increase the key rates. Third, in our experiment, we utilized several manual Fiber Polarization Controllers (FPCs) which were needed to maintain the polarization entangled state at each stage of the multiplexing, demultiplexing, beamsplitting and distribution. For expediency and to demonstrate the success of our network topology, we considered it sufficient when each of the FPCs were aligned with $> 97\,\%$ visibility. A better fiber neutralization would have resulted in improved key rates. Lastly, in an attempt to keep the costs of each user's Polarization Analysis Module (PAM) to a minimum, we used readily available sub-par components which we estimate contribute to the overall QBER by up to an additional $1\,\%$.

Security considerations: In this section, we provide a more detailed analysis of the security of the implemented protocol. The protocol used in our experiment is slightly different from the original BBM92 in the following aspects. First, while the random choice of measurement basis is performed passively in our setup by a 50:50 beamsplitter, the users cannot tell in which basis they have done the measurement. Instead, if after correcting for their time offsets, they both detect a photon within a coincidence window of width τ_c, they assume that they have both used the same basis. In this mixed-basis case (34), the estimation of the phase error rate from the observed bit error rate must be done with caution. In particular, because, in our setup, the employed beamsplitters in the receiver units may not be exactly 50:50, one should account for its effect on the secret key rate.

Here, we use a simplified picture of the protocol to account for the above two issues in our security analysis. We only consider two nominal users, Alice and Bob; the same argument holds for any pair of users in our setup. Without loss of generality, we assume that Alice and Bob share the same time reference and that the transmission delay between the source and each of the two users is zero. Now consider a particular pattern of detection events that corresponds to a certain sifted key bit. That is, suppose Alice and Bob have detection events, respectively, at time t_A and t_B such that $|t_A - t_B| < \tau_c/2$. We refer to such an event as a coincidence with time offset at the receiver, Δ_{Rx}, equal to zero. (More generally, we have a coincidence event at a nonzero Δ_{Rx} if $|t_A - t_B - \Delta_{\mathrm{Rx}}| < \tau_c/2$.) There should then be a transmitted signal to Alice (Bob)

at time $\tau_{A(B)} \in \{t_{A(B)}, t_{A(B)} - \Delta\}$, where Δ is the time difference between the long and short optical paths, used for X and Z basis measurements, in the PAMs. For simplicity, we assume the time delay in the short path is zero.

The key point in our security proof is that, so long as $\Delta \gg \tau_c$, the only detection events that can be used for secure key extraction are those for which $|\tau_A - \tau_B| < \tau_c/2$. We refer to such an event as a coincidence with time offset at the transmitter, Δ_{Tx}, equal to zero. More generally, we have a coincidence event at a nonzero Δ_{Tx} if $|\tau_A - \tau_B - \Delta_{\text{Tx}}| < \tau_c/2$. As we explain below, the detection events that originate from transmitted signals with $\Delta_{\text{Tx}} = \pm\Delta$ can easily be manipulated by a potential eavesdropper to give us insecure detection events. Note that in the trust-free QKD setting that we are considering, we cannot assume that the source is trustworthy. Even if we make this assumption, an eavesdropper can block the trusted source output, and, instead, send her own signals to the users. Now, imagine such an eavesdropper is sending an A-polarized photon to Alice at time $t_A - \Delta$ and an H-polarized photon to Bob at time t_B, with $|t_A - t_B| < \tau_c/2$. Then a detection event at times t_A and t_B would correspond to the same bit but different bases, while Alice and Bob would falsely assume that these are in the same basis as expected from an honest source. However, the eavesdropper can tell without any error the bits assigned to the sifted key in such a case. In other words, in the terminology of the GLLP analysis (35), these bits are *tagged*.

Luckily, such an eavesdropping attempt would leave a footprint, which could be used to estimate the amount of information that has leaked to Eve. In the

example above, a signal generated at $t_A - \Delta$ may also take the shorter path and cause a click at the same time on Alice's side, while the signal generated at t_B takes the longer path and causes a click at $t_B + \Delta$. Having a coincidence event with a time offset $\Delta_{\mathrm{Rx}} = 2\Delta$ would not have been expected if the signals sent to Alice and Bob are generated at the same time. We will use the collected data on the latter events to bound the number of tagged sifted key bits.

Based on the above, for any coincident event with $\Delta_{\mathrm{Rx}} = 0$, there are only three possible transmission time offsets, namely, $\Delta_{\mathrm{Tx}} \in \{-\Delta, 0, \Delta\}$. The sifted key bits for which Eve chooses $\Delta_{\mathrm{Tx}} = \pm\Delta$ are tagged: We should assume that Eve can fully learn them without introducing any errors. The sifted key bits for which Eve chooses $\Delta_{\mathrm{Tx}} = 0$ are untagged, and they can be regarded as having arisen from an execution of a standard BBM92 in which Alice and Bob have been able to postselect the detected rounds in which they have used the same basis, but not to learn their specific choice of basis for each round. We assume that the signals received in these rounds are in a qubit space (i.e., a polarized single photon); if they are not, one can still prove security by using the techniques of (36, 37) and assigning a random sifted bit to events in which more than one detector clicks in a particular round. To estimate the amount of secret key that can be extracted, we then need to obtain: **(1)** a lower bound on the number of untagged bits $N_{0,0}$ in the sifted key, where $N_{R,T}$ denotes the number of events in which $\Delta_{\mathrm{Rx}} = R$ and $\Delta_{\mathrm{Tx}} = T$; and **(2)** an upper bound on the phase error rate of these bits, which we denote as e_p.

(1) Lower bound on $N_{0,0}$: For bounding $N_{0,0}$, we use the fact that when Eve

chooses $\Delta_{\text{Tx}} = \pm\Delta$, the probability of having $\Delta_{\text{Rx}} = 0$ is the same as that of $\Delta_{\text{Rx}} = \pm 2\Delta$. We denote the number of coincidence events with receiver time offsets of either zero or $\pm 2\Delta$ by N. For event n out of these N events, we then have

$$\Pr[\Delta_{\text{Tx}}^{(n)} \in \{\Delta, -\Delta\}, \Delta_{\text{Rx}}^{(n)} = 0] = \Pr[\Delta_{\text{Tx}}^{(n)} \in \{\Delta, -\Delta\}, \Delta_{\text{Rx}}^{(n)} \in \{2\Delta, -2\Delta\}] \le \Pr[\Delta_{\text{Rx}}^{(n)} \in \{2\Delta, -2\Delta\}], \tag{4}$$

where the superscript (n) specifies the value of the time offset parameters for the nth event. By Azuma's inequality, we have that

$$\begin{aligned} \sum_{n=1}^{N} \Pr[\Delta_{\text{Tx}}^{(n)} \in \{\Delta, -\Delta\}, \Delta_{\text{Rx}}^{(n)} = 0] \ge N_{0,\Delta} + N_{0,-\Delta} - \delta, \\ \sum_{n=1}^{N} \Pr[\Delta_{\text{Rx}}^{(n)} =\in \{2\Delta, -2\Delta\}] \le N_{2\Delta} + N_{-2\Delta} + \delta, \end{aligned} \tag{5}$$

where each of the bounds fails with probability ε, $\delta = \sqrt{2N \ln \varepsilon^{-1}}$ is the deviation term, and N_R is the total number of detections for which $\Delta_{\text{Rx}} = R$. The conditioning on the outcome of the previous detections has been omitted from all probability terms for simplicity. Combining Eq. (4) and Eq. (5), we have that

$$N_{0,\Delta} + N_{0,-\Delta} \le N_{2\Delta} + N_{-2\Delta} + 2\delta, \tag{6}$$

and, therefore,

$$N_{0,0} = N_0 - N_{0,\Delta} - N_{0,-\Delta} \ge N_0 - N_{2\Delta} - N_{-2\Delta} - 2\delta := N_{0,0}^{(L)}, \tag{7}$$

except with probability 2ε.

(2) Upper bound on e_p: To bound the phase-error rate of the untagged bits (that is, those bits for which $\Delta_{\text{Tx}} = 0$ and $\Delta_{\text{Rx}} = 0$), we bound the number of

phase errors N_{ph} that Alice and Bob would have obtained in a hypothetical scenario in which they have made exactly the opposite basis choices as in the real scenario. Let us assume that $p_Z^A = p_Z^B = p_X^A = p_X^B = \frac{1}{2}$, where $p_K^{A(B)}$ is the probability of choosing basis $K = X, Z$ by Alice (Bob). Then, the probability that Alice and Bob both measure in the Z (X) basis in the real (hypothetical) scenario is the same as the probability that they both measure in the X (Z) basis in the real (hypothetical) scenario. In this situation, we have that, for a given untagged round, the probability that Alice and Bob obtain an error is the same for both the real and hypothetical scenarios. That is, its bit error probability equals its phase error probability (34).

If the measurement basis choice is biased, the two are no longer necessarily equal. Say that Alice and Bob are α times as likely to jointly choose one basis than the other, e.g. $p_Z^A p_Z^B = \alpha p_X^A p_X^B$ with $\alpha \geq 1$. Then, if Eve makes the X-basis error probability larger than the Z-basis error probability, the phase-error probability will be larger than the bit-error probability. Still, one can easily show that the phase-error probability will be at most α times larger than the bit-error probability (34). Then, we have that

$$\begin{aligned} \Pr[\Delta_{\mathrm{Tx}}^{(n)} = 0, \Delta_{\mathrm{Rx}}^{(n)} = 0, \text{phase error}] &\leq \alpha \Pr[\Delta_{\mathrm{Tx}}^{(n)} = 0, \Delta_{\mathrm{Rx}}^{(n)} = 0, \text{bit error}] \\ &\leq \alpha \Pr[\Delta_{\mathrm{Rx}}^{(n)} = 0, \text{bit error}]. \end{aligned} \tag{8}$$

And, by Azuma's inequality, we have that

$$\begin{aligned} \sum_{n=1}^{N} \Pr[\Delta_{\mathrm{Tx}}^{(n)} = 0, \Delta_{\mathrm{Rx}}^{(n)} = 0, \text{phase error}] \geq N_{\mathrm{ph}} - \delta, \\ \sum_{n=1}^{N} \Pr[\Delta_{\mathrm{Rx}}^{(n)} = 0, \text{bit error}] \leq N_{\mathrm{err}} - \delta, \end{aligned} \tag{9}$$

where N_{err} is the amount of bits in the sifted key that have a bit error (i.e. $e_b = \frac{N_{\text{err}}}{N_0}$), and N_{ph} is the number of phase errors, defined above. Combining Eq. (8) and Eq. (9), we have that

$$N_{\text{ph}} \leq \alpha N_{\text{err}} + (1+\alpha)\delta := N_{\text{ph}}^{(U)}, \tag{10}$$

except with probability 2ε. The phase-error rate can now be simply upper-bounded by

$$e_p^U = \frac{N_{\text{ph}}^{(U)}}{N_{0,0}^{(L)}}. \tag{11}$$

Finally, the length of secret key that can be distilled is given by

$$n_f \geq N_{0,0}^{(L)} \left[1 - H_2(e_p^U)\right] - f(e_b) N_0 H_2(e_b). \tag{12}$$

In the methods section, we use a simpler version of the above expression in which we assume that $\Delta_{\text{Tx}} = 0$ for all rounds. In this case, all sifted-key bits are untagged, which implies that $N_{0,0}^{(L)} = N_{0,0} = N_0 \equiv n_s$, and e_p^U in Eq. (11) reduces to Eq. (3) of the main text, with $\xi_{ph} = 2\varepsilon$.

	Bob	Chloe	Dave	Feng	Gopi	Heidi	Ivan
Alice	10.03	10.08	9.58	13.37	16.53	9.06	6.81
Bob		9.14	8.58	17.32	6.24	7.44	5.95
Chloe			14.25	12.64	7.01	8.67	14.63
Dave				10.64	6.33	20.27	11.45
Feng					9.01	10.80	4.44
Gopi						5.96	3.88
Heidi							6.43

Table S1: **Total secure key (Mega bits) for the laboratory demonstration** as measured continuously over 18.45 hours after accounting for all finite key size effects.

	Bob	Chloe	Dave	Feng	Gopi	Heidi	Ivan
Alice	31143	8926	6087	15590	38075	6637	901
Bob		23747	24171	83986	41239	9380	9787
Chloe			16850	14842	17910	9511	12694
Dave				1516	17004	14230	4356
Feng					20121	10142	810
Gopi						9954	3759
Heidi							1747

Table S2: **Total secure key (bits) over long distance links for the city wide metropolitan network demonstration.** We connected 4 locations/users across the city of Bristol as shown in Fig. 2 via deployed fiber in a loop back configuration. Two other users were sent signals through fiber spools and the remaining two were connected via short (10 m) fibers. The distances of all 28 links are given in Table **S3**. Considering finite-size-effects, we measured for ~27 minutes to obtain the final secure key shown. Here, we set the failure probability of phase error estimation to 10^{-5}. Fig. **S4** shows the overall stability of the key for 7 hours.

	Bob	Chloe	Dave	Feng	Gopi	Heidi	Ivan
Alice	12642	13095	16971	14257	12642	14256	15735
Bob		473	4350	1636	20	1634	3113
Chloe			4803	2089	473	2087	3566
Dave				5965	4350	5963	7442
Feng					1636	3250	4728
Gopi						1635	3113
Heidi							4727

Table S3: **Length of each link in meters** in the metropolitan network shown in meters. Bob and Gopi were users separated by 10 m of fiber each from the QNSP. Alice and Dave were connected to ~12.6 km and ~4.3 km spools of fiber. The remaining users were connected via loop-back to various locations across the city of Bristol as shown in Fig. 2. Each link was characterized by an OTDR and the measurements shown are the link distances in fiber between each pair of users.

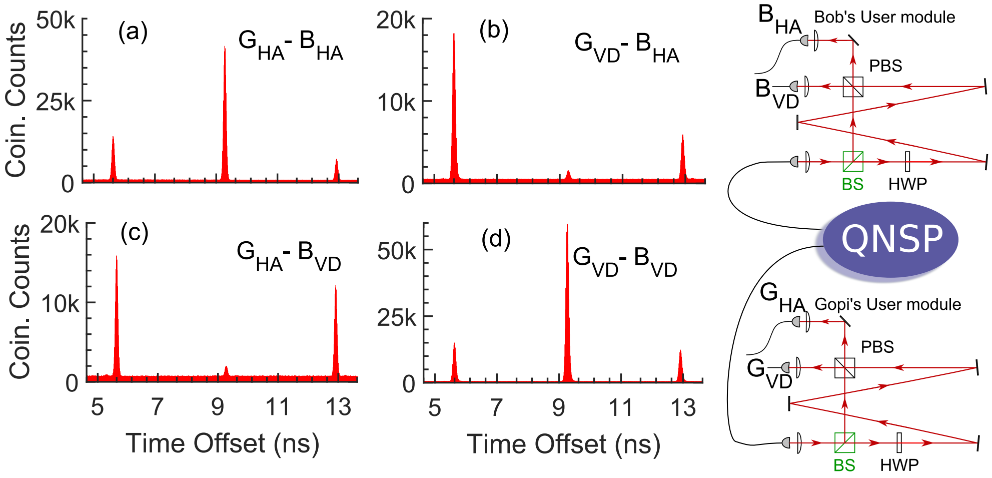

Fig. S1: **Temporal cross correlation histograms between users Gopi and Bob illustrate how users generate quantum secure keys**: $g^{(2)}$ histograms between users Gopi (G) and Bob (B) are shown without obscuring the channel information. Each user's Polarization Analysis Module (PAM) detects photons in the Horizontal (H) or Anti-diagonal (A) basis on detector 1 and Vertical (V) or Diagonal (D) on detector 2. D and A detection events are delayed with respect to H and V by ~3.7 ns. Four different histograms corresponding to each possible pair of detectors between G and B are shown. From this data we can directly measure the QBER by comparing the desired middle peaks (upper left & lower right) with the undesired ones (upper right & lower left). The data shown was integrated over one hour. To the right is a simplified schematic showing two users connected to the Quantum Network Service Provider (QNSP) with the relevant detectors labeled. The peak separation of 3.7 ns is primarily due to the optical path length difference is each user module.

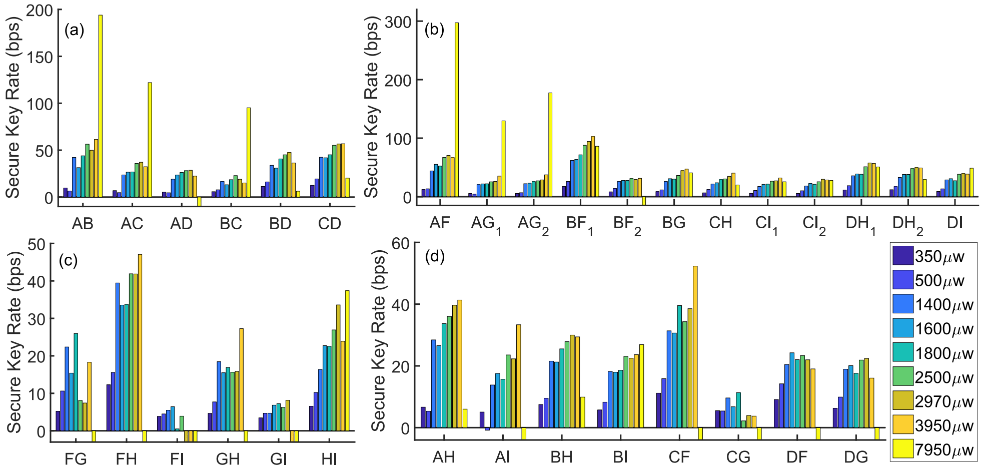

Fig. S2: **Optimizing the average secure key rate by adjusting the pump power**: The amount of secure key obtained per second can be optimized by increasing the pump power at the source and hence the pair production rate.

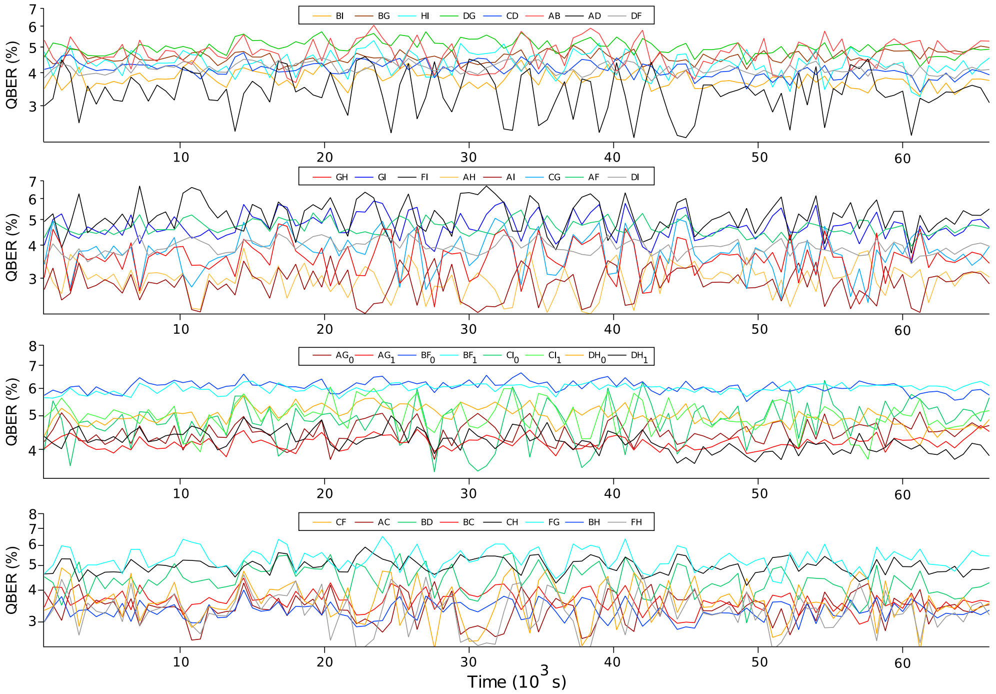

Fig. S3: **Stability of the QBER over extended periods of time proves that polarisation encoding over fibers is a viable solution**. We tested the passive stability of our network over short links and a very long time of 18.45 hours. The QBER for each pair of users is shown here. In addition, those users with premium links (i.e. more than one set of correlated wavelengths) shared between them have two independent values of the QBER and are indicated using the subscripts 0,1. The secure key rate for this measurement is shown in Fig. 3.

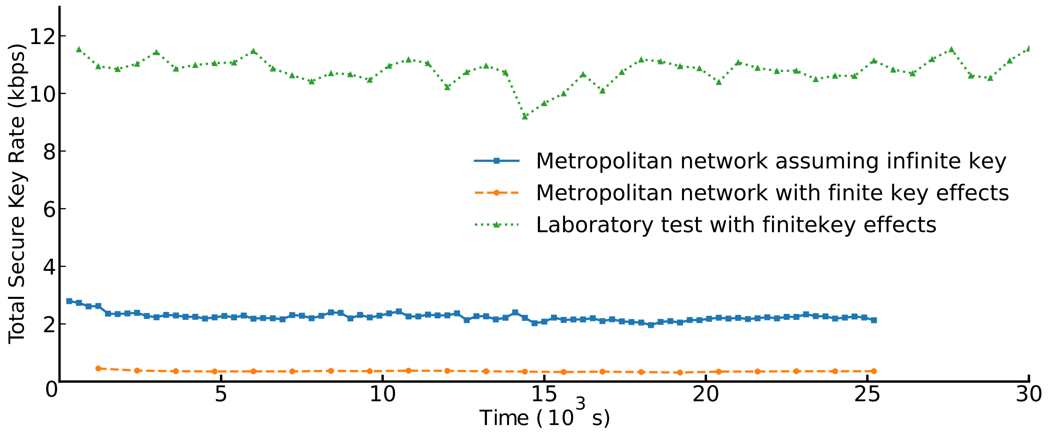

Fig. S4: **Stability of the secure key rate over time**. To compare the test network in the laboratory with the real world deployed citywide fiber network, we summed up the key rates from each pair of users. We note that despite the high losses and large distances involved (up to ~17 km) the network's key rate remains stable. The key rate in bits per second is shown while considering finite key effects for the Metropolitan quantum communication network (laboratory test) in blue solid line (green dotted line) using block sizes of 20 min (10 min). For comparison we also show the key rate of the city wide network assuming an infinite key length averaged over a block size of 5 min.

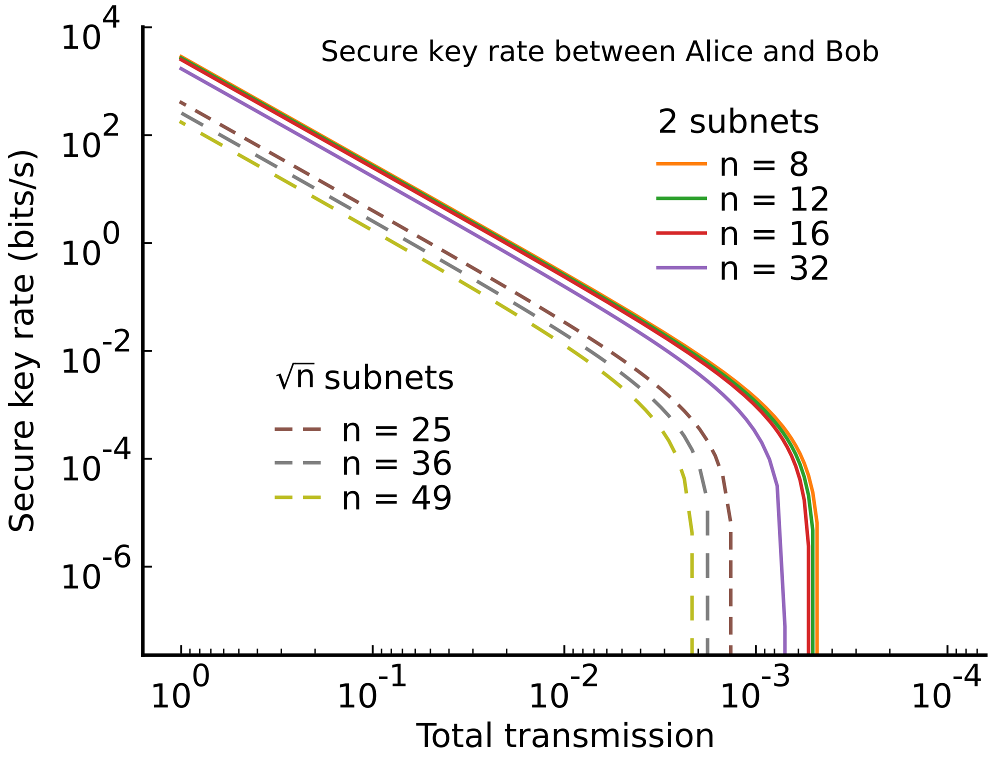

Fig. S5: **Simulation showing the scalability of the current network topology**. The graph shows the secure key rate between two users who do not share a premium link. The simulation used detectors with 100 ps jitter and 70 % detection efficiency. Further, the source was assumed to produce 10^5 pairs per second per correlated wavelength pair with a heralding efficiency of 20 %. The **solid lines** represent topologies with n users split between 2 subnets using only 2-fold beamsplitters. **The dashed lines** represent the use of $\sqrt{n}$-fold beamsplitters to create $\sqrt{n}$ subnets. 128 wavelength channels are needed for the $n = 32$ 2-subnet topology, while only 84 wavelength channels are needed for the $n = 49$ 7-subnet topology. The reduced key rates of the $\sqrt{n}$-subnet topologies, despite having fewer wavelength channels, is due to the loss introduced by the $\sqrt{n}$-fold beamsplitters.

REFERENCES AND NOTES

1. V. Scarani, H. Bechmann-Pasquinucci, N. J. Cerf, M. Dušek, N. Lütkenhaus, M. Peev, The security of practical quantum key distribution. *Rev. Mod. Phys.* **81**, 1301 (2009).

2. S. Pirandola, U. L. Andersen, L. Banchi, M. Berta, D. Bunandar, R. Colbeck, D. Englund, T. Gehring, C. Lupo, C. Ottaviani, J. Pereira, M. Razavi, J. S. Shaari, M. Tomamichel, V. C. Usenko, G. Vallone, P. Villoresi, P. Wallden, Advances in quantum cryptography. arXiv:1906.01645 (2019).

3. S. Wengerowsky, S. K. Joshi, F. Steinlechner, J. R. Zichi, S. M. Dobrovolskiy, R. van der Molen, J. W. N. Los, V. Zwiller, M. A. M. Versteegh, A. Mura, D. Calonico, M. Inguscio, H. Hübel, L. Bo, T. Scheidl, A. Zeilinger, A. Xuereb, R. Ursin, Entanglement distribution over a 96-km-long submarine optical fiber. *Proc. Natl. Acad. Sci. U.S.A.* **116**, 6684–6688 (2019).

4. F. Hipp, M. Hentschel, S. Aleksic, A. Poppe, H. Huebel, Demonstration of a coexistence scheme between polarization-entangled QKD and classical data channels, in *Quantum Optics* (International Society for Optics and Photonics, 2016), vol. 9900, p. 99000P.

5. L.-J. Wang, L.-K. Chen, L. Ju, M.-L. Xu, Y. Zhao, K. Chen, Z.-B. Chen, T.-Y. Chen, J.-W. Pan, Experimental multiplexing of quantum key distribution with classical optical communication. *Appl. Phys. Lett.* **106**, 081108 (2015).

6. K. A. Patel, J. F. Dynes, I. Choi, A. W. Sharpe, A. R. Dixon, Z. L. Yuan, R. V. Penty, A. J. Shields, Coexistence of high-bit-rate quantum key distribution and data on optical fiber. *Phys. Rev. X* **2**, 041010 (2012).

7. J. Qiu, Quantum communications leap out of the lab. *Nature* **508**, 441–442 (2014).

8. S. Wengerowsky, S. K. Joshi, F. Steinlechner, J. R. Zichi, B. Liu, T. Scheidl, S. M. Dobrovolskiy, R. van der Molen, J. W. N. Los, V. Zwiller, M. A. M. Versteegh, A. Mura, D. Calonico, M. Inguscio, A. Zeilinger, A. Xuereb, R. Ursin, Passively stable distribution of polarisation entanglement over 192 km of deployed optical fibre. *npj Quantum Inf.* **6**, 5 (2020).

9. M. Peev, C. Pacher, R. Alléaume, C. Barreiro, J. Bouda, W. Boxleitner, T. Debuisschert, E. Diamanti, M. Dianati, J. F. Dynes, S. Fasel, S. Fossier, M. Fürst, J.-D. Gautier, O. Gay, N. Gisin, P. Grangier, A. Happe, Y. Hasani, M. Hentschel, H. Hübel, G. Humer, T. Länger, M. Legré, R. Lieger, J. Lodewyck, T. Lorünser, N. Lütkenhaus, A. Marhold, T. Matyus, O. Maurhart, L. Monat, S. Nauerth, J.-B. Page, A. Poppe, E. Querasser, G. Ribordy, S. Robyr, L. Salvail, A. W. Sharpe, A. J. Shields, D. Stucki, M. Suda, C. Tamas, T. Themel, R. T. Thew, Y. Thoma, A. Treiber, P. Trinkler, R. Tualle-Brouri, F. Vannel, N. Walenta, H. Weier, H. Weinfurter, I. Wimberger, Z. L. Yuan, H. Zbinden, A. Zeilinger, The SECOQC quantum key distribution network in Vienna. *New J. Phys.* **11**, 075001 (2009).

10. M. Sasaki, M. Fujiwara, H. Ishizuka, W. Klaus, K. Wakui, M. Takeoka, S. Miki, T. Yamashita, Z. Wang, A. Tanaka, K. Yoshino, Y. Nambu, S. Takahashi, A. Tajima, A. Tomita, T. Domeki, T. Hasegawa, Y. Sakai, H. Kobayashi, T. Asai, K. Shimizu, T. Tokura, T. Tsurumaru, M. Matsui, T. Honjo, K. Tamaki, H. Takesue, Y. Tokura, J. F. Dynes, A. R. Dixon, A. W. Sharpe, Z. L. Yuan, A. J. Shields, S. Uchikoga, M. Legré, S. Robyr, P. Trinkler, L. Monat, J.-B. Page, G. Ribordy, A. Poppe, A. Allacher, O. Maurhart, T. Länger, M. Peev, A. Zeilinger, Field test of quantum key distribution in the Tokyo QKD Network. *Opt. Express* **19**, 10387–10409 (2011).

11. D. Stucki, M. Legré, F. Buntschu, B. Clausen, N. Felber, N. Gisin, L. Henzen, P. Junod, G. Litzistorf, P. Monbaron, L. Monat, J.-B. Page, D. Perroud, G. Ribordy, A. Rochas, S. Robyr, J. Tavares, R. Thew, P. Trinkler, S. Ventura, R. Voirol, N. Walenta, H. Zbinden, Long-term performance of the SwissQuantum quantum key distribution network in a field environment. *New J. Phys.* **13**, 123001 (2011).

12. F. Xu, W. Chen, S. Wang, Z. Q. Yin, Y. Zhang, Y. Liu, Z. Zhou, Y. B. Zhao, H. W. Li, D. Liu, Z. F. Han, G. C. Guo, Field experiment on a robust hierarchical metropolitan quantum cryptography network. *Chin. Sci. Bull.* **54**, 2991–2997 (2009).

13. S. Wang, W. Chen, Z.-Q. Yin, H.-W. Li, D.-Y. He, Y.-H. Li, Z. Zhou, X.-T. Song, F.-Y. Li, D. Wang, H. Chen, Y.-G. Han, J.-Z. Huang, J.-F. Guo, P.-L. Hao, M. Li, C.-M. Zhang, D. Liu, W.-Y. Liang, C.-H. Miao, P. Wu, G.-C. Guo, Z.-F. Han, Field and long-term demonstration of a wide area quantum key distribution network. *Opt. Express* **22**, 21739–21756 (2014).

14. P. Toliver, P. Toliver, R. J. Runser, T. E. Chapuran, J. L. Jackel, T. C. Banwell, M. S. Goodman, R. J. Hughes, C. G. Peterson, D. Derkacs, J. E. Nordholt, L. Mercer, S. Mc Nown, A. Goldman, J. Blake, Experimental investigation of quantum key distribution through transparent optical switch elements. *IEEE Photon. Technol. Lett.* **15**, 1669–1671 (2003).

15. T.-Y. Chen, J. Wang, H. Liang, W.-Y. Liu, Y. Liu, X. Jiang, Y. Wang, X. Wan, W.-Q. Cai, L. Ju, L.-K. Chen, L.-J. Wang, Y. Gao, K. Chen, C.-Z. Peng, Z.-B. Chen, J.-W. Pan, Metropolitan all-pass and inter-city quantum communication network. *Opt. Express* **18**, 27217–27225 (2010).

16. C. Elliott, A. Colvin, D. Pearson, O. Pikalo, J. Schlafer, H. Yeh, Current status of the DARPA quantum network, in *Quantum Information and Computation III*, E. J. Donkor, A. R. Pirich, H. E. Brandt, Eds. (International Society for Optics and Photonics, SPIE, 2005), vol. 5815, pp. 138–149.

17. X.-Y. Chang, D.-L. Deng, X.-X. Yuan, P.-Y. Hou, Y.-Y. Huang, L.-M. Duan, Experimental realization of an entanglement access network and secure multi-party computation. *Sci. Rep.* **6**, 29453 (2016).

18. S. Wengerowsky, S. K. Joshi, F. Steinlechner, H. Hübel, R. Ursin, An entanglement-based wavelength-multiplexed quantum communication network. *Nature* **564**, 225–228 (2018).

19. I. Herbauts, B. Blauensteiner, A. Poppe, T. Jennewein, H. Hübel, Demonstration of active routing of entanglement in a multi-user network. *Opt. Express* **21**, 29013–29024 (2013).

20. P. D. Townsend, Quantum cryptography on multiuser optical fibre networks. *Nature* **385**, 47–49 (1997).

21. I. Choi, R. J. Young, P. D. Townsend, Quantum information to the home. *New J. Phys.* **13**, 063039 (2011).

22. B. Fröhlich, J. F. Dynes, M. Lucamarini, A. W. Sharpe, Z. Yuan, A. J. Shields, A quantum access network. *Nature* **501**, 69–72 (2013).

23. D. Aktas, B. Fedrici, F. Kaiser, T. Lunghi, L. Labonté, S. Tanzilli, Entanglement distribution over 150 km in wavelength division multiplexed channels for quantum cryptography. *Laser Photon. Rev.* **10**, 451–457 (2016).

24. E. Y. Zhu, C. Corbari, A. V. Gladyshev, P. G. Kazansky, H.-K. Lo, L. Qian, Multi-party agile QKD network with a fiber-based entangled source, in *2015 Conference on Lasers and Electro-Optics (CLEO)* (Optical Society of America, 2015), pp. 1–2.

25. H. C. Lim, A. Yoshizawa, H. Tsuchida, K. Kikuchi, Broadband source of telecom-band polarization-entangled photon-pairs for wavelength-multiplexed entanglement distribution. *Opt. Express* **16**, 16052–16057 (2008).

26. P. Törmä, K. M. Gheri, Establishing multi-party entanglement with entangled photons, in *Mysteries, Puzzles, and Paradoxes in Quantum Mechanics* (ASCE, 1999), pp. 220–228.

27. M. Pivoluska, M. Huber, M. Malik, Layered quantum key distribution. *Phys. Rev. A* **97**, 032312 (2018).

28. C. H. Bennett, G. Brassard, N. D. Mermin, Quantum cryptography without Bell's theorem. *Phys. Rev. Lett.* **68**, 557–559 (1992).

29. E. Y. Zhu, C. Corbari, A. Gladyshev, P. G. Kazansky, H.-K. Lo, L. Qian, Toward a reconfigurable quantum network enabled by a broadband entangled source. *J. Opt. Soc. Am. B* **36**, B1–B6 (2019).

30. C. Chen, A. Riazi, E. Y. Zhu, M. Ng, A. V. Gladyshev, P. G. Kazansky, L. Qian, Turn-key diode-pumped all-fiber broadband polarization-entangled photon source. *OSA Continuum* **1**, 981–986 (2018).

31. T. Kim, M. Fiorentino, F. N. C. Wong, Phase-stable source of polarization-entangled photons using a polarization Sagnac interferometer. *Phys. Rev. A* **73**, 012316 (2006).

32. A. Fedrizzi, T. Herbst, A. Poppe, T. Jennewein, A. Zeilinger, A wavelength-tunable fiber-coupled source of narrowband entangled photons. *Opt. Express* **15**, 15377–15386 (2007).

33. C.-H. F. Fung, X. Ma, H. F. Chau, Practical issues in quantum-key-distribution postprocessing. *Phys. Rev. A* **81**, 012318 (2010).

34. O. Gayer, Z. Sacks, E. Galun, A. Arie, Temperature and wavelength dependent refractive index equations for MgO-doped congruent and stoichiometric $LiNbO_3$. *Appl. Phys. B.* **91**, 343–348 (2008).

35. D. Gottesman, H.-K. Lo, N. Lutkenhaus, J. Preskill, Security of quantum key distribution with imperfect devices, in *International Symposium on Information Theory, 2004. ISIT 2004. Proceedings* (IEEE, 2004), p. 136.

36. T. Tsurumaru, K. Tamaki, Security proof for quantum-key-distribution systems with threshold detectors. *Phys. Rev. A* **78**, 032302 (2008).

37. N. J. Beaudry, T. Moroder, N. Lütkenhaus, Squashing models for optical measurements in quantum communication. *Phys. Rev. Lett.* **101**, 093601 (2008).
